# Photoswitching Lanthanoid Luminescence With Diazocines

**DOI:** 10.1002/chem.202500397

**Published:** 2025-04-21

**Authors:** Nicole Winterholler, Elisabeth Kreidt

**Affiliations:** ^1^ Department of Chemistry and Chemical Biology TU Dortmund University Otto‐Hahn‐Str. 6 Dortmund 44227 Germany

**Keywords:** azo‐compounds, lanthanide, luminescence, photochromism

## Abstract

The unique photophysical properties of the lanthanoids provide the core functionality for various high‐tech applications, including optoelectronic materials, lasers, anti‐counterfeiting dyes, or tools for biological sciences. The combination of these luminophores with photoswitches has the potential to make them remote controllable and adds new layers of complexity to their applications. We herein report that such a photomodulation of Eu^III^, Tb^III^, and Yb^III^ luminescence is possible with the aid of diazocine photoswitches. The new approach, which does not require covalent connection of the photoswitch and antenna ligand, is by design suitable for multiplexing applications and allows for a drastic and reversible modulation of the overall luminescence intensity and luminescence lifetimes. Photophysical studies under bypassing of the antenna ligand and at 77 K gave insights into the mechanism of the effect.

## Graphical abstract

Photomodulation of lanthanoid luminescence promises new layers of complexity for diverse applications. We herein present a new strategy for the manipulation of luminescence intensities and lifetimes based on diazocine photoswitches. This strategy is particularly well suited for multiplexing and does not require covalent connection of individual photoactive components.

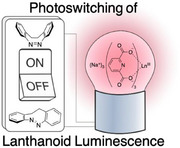

## 1 Introduction

In the development of smart optical materials, photoswitches are key building blocks. Upon irradiation with light of specific wavelengths, they change their geometry and electronic structure, allowing for the reversible remote control of microscopic as well as macroscopic properties with excellent spatio–temporal resolution.^[^ ^1^ ^]^ A particularly interesting case are smart luminophores in which photoswitches are used to reversibly manipulate a luminescence response or read‐out, for example in bioimaging, anti‐counterfeiting or data storage.^[^ ^2^ ^]^

Luminescent complexes of trivalent lanthanoids (Ln^III^) are a special class of luminophores with unique properties.^[^ ^3^ ^]^ The highly characteristic emission spectra of the lanthanoids, consisting of narrow bands, allow for unambiguous identification and quantification, even in samples containing several emissive lanthanoids, as is required in optical multiplexing applications.^[^ ^4^ ^]^ Together with their long luminescence lifetimes, up to the millisecond range for Eu^III^ and Tb^III^, and the availability of NIR‐emitting lanthanoids such as Yb^III^, this makes lanthanoids ideal candidates as emissive components in all‐optical smart luminophores. There are already some examples in which photoswitches have successfully been used to control the luminescence intensities and lifetimes of lanthanoid luminophores.^[^ ^5^ ^]^ The pioneering and still most commonly applied strategy is the use of diarylethenes (DAEs) for modification of Eu^III^ luminescence.^[^ ^6^ ^]^ Examples with alternative photoswitches are still relatively sparse and include systems based on spirobenzopyran,^[^ ^7^ ^]^ azobenzene,^[^ ^8^ ^]^ a photoreaction of anthrancene,^[^ ^9^ ^]^ or a boryl chromophore,^[^ ^10^ ^]^ the latter two requiring thermal backswitching. Apart from Eu^III^, a few examples in which the luminescence of Tb^III[^ ^8^, ^11^ ^]^ or Yb^III[^ ^6^, ^12^ ^]^ (or Er^III[^ ^13^ ^]^ in the solid state) was photoswitched have been reported; however, up until now, for none of the described systems photomodulation of the luminescence of more than two different Ln^III[^ ^6^, ^8^, ^11^, ^12^ ^]^ has been reported, which would be a prerequisite for any multiplexing application.

**Figure 1:**
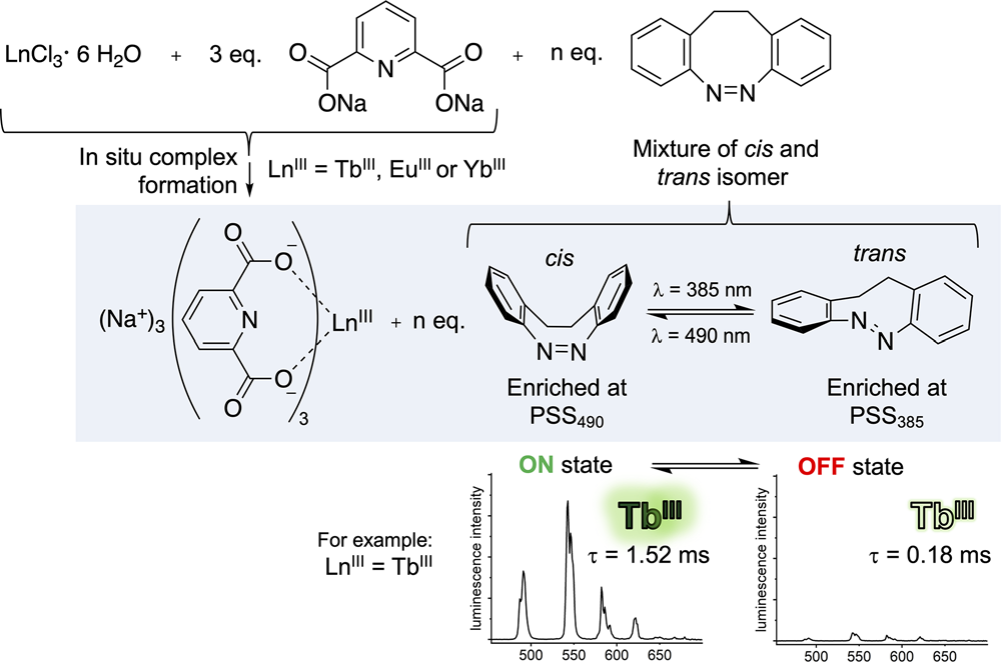
Preparation and composition of the samples studied herein and the effect of diazocine photoswitching upon Tb^III^ emission. PSS_490_ and PSS_385_ refer to the photostationary states (PSS) obtained after irradiation with light of wavelengths *λ* = 490 nm or *λ* = 385 nm, respectively.

Herein we report a new strategy for the photomodulation of lanthanoid luminescence tailored to overcome this limitation. Using simple diazocine photoswitches, we could reversibly manipulate the luminescence intensities and lifetimes of lanthanoid dipicolinates Na_3_[Ln(DPA)_3_] with Ln = Tb^III^, Eu^III^, and Yb^III^. We observed effects of up to 93% reversible reduction of luminescence intensity and a shortening of luminescence lifetimes by over a millisecond. Our approach does not require covalent connection of the photoactive components and instead utilizes simple, well‐accessible building blocks (see Figure 1).

**Figure 2:**
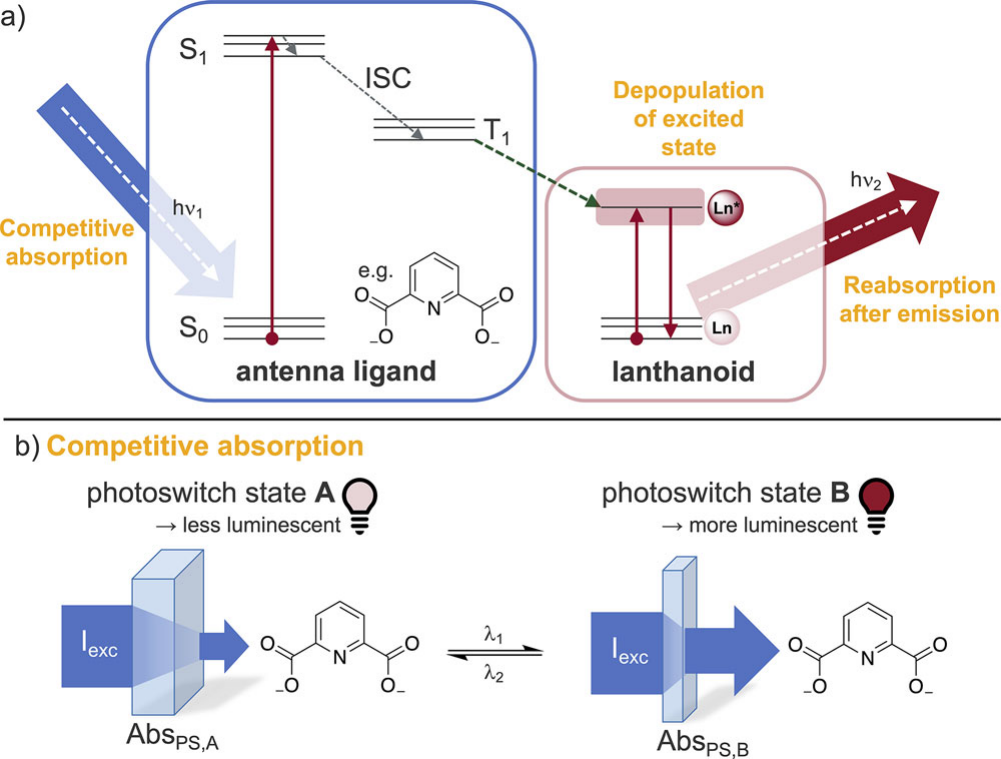
Indirect sensitization of lanthanoid luminophores and resulting potential mechanisms for luminescence photoswitching. a) Jablonski diagram of the antenna effect. Upon irradiation the antenna ligand (shown here is the exemplary dipicolinic acid antenna) is brought to an excited state S_1_. Intersystem crossing (ISC) populates a T_1_ state from which the energy is transferred upon the lanthanoid (green dashed arrow). b) Luminescence photomodulation via competitive absorption. Abs_PS,A_ and Abs_PS,B_ refer to the switching state absorptivity of the photoswitch. See main text for details.

Due to the forbidden nature of the f–f transitions of the trivalent lanthanoids, they have very small extinction coefficients. Hence, in lanthanoid luminescence, typically indirect sensitization with the aid of an organic chromophore (“antenna”) in close proximity to the lanthanoid is employed. In the overall process leading to lanthanoid luminescence, initially the antenna is excited into an excited singlet state by irradiation with light of a suitable wavelength; via intersystem crossing (ISC), then a long‐lived triplet state is populated before the energy is transferred to the lanthanoid (Figure 2a).^[^ ^3^, ^14^ ^]^

This overall scheme provides several processes and states as potential targets for manipulation by a photoswitch. However, the most suitable to employ in a multiplexing–compatible strategy is the initial absorption of photons (see Figure 2b) by the antenna. If the photoswitch has a switching state‐dependent absorbance at the wavelength used for antenna excitation, this can be utilized to dynamically modify the number of photons available for the overall antenna process (“competitive absorption”). In this case the photoswitch manipulates the overall luminescence intensity almost like a (modifiable) optical filter placed in front of the sample. One of the most beneficial side effects of utilization of the antenna effect in lanthanoid luminescence is that in principle various luminescent lanthanoids can be sensitized with the same antenna, i.e., via the same excitation wavelength. An important example is the dipicolinic acid anion (DPA^2‐^), which upon UV irradiation (*λ* = 271 nm) is able to sensitize Eu^III^, Tb^III^, Sm^III^, Dy^III^, Yb^III^, and Nd^III^.^[^ ^15^ ^]^ Since the competitive absorption targets the very initial absorption of light by the antenna ligand (which is independent of the lanthanoid), with a given pair of an antenna and a suitable photoswitch, the competitive absorption strategy for luminescence photomodulation should in principle work for any lanthanoid compatible with the antenna, without further adaptions of the system.

## 2 Results And Discussion

### 2.1 Design of Study

**Figure 3:**
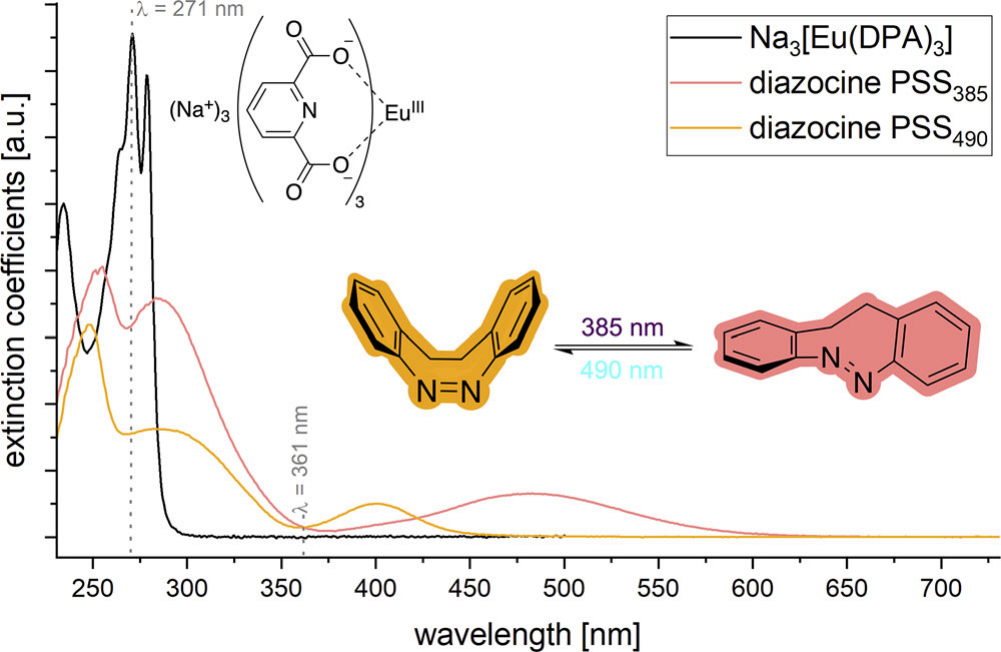
Structures of diazocine in both isomeric forms with absorption spectra of the respective photostationary states and absorption spectrum of Na_3_[Eu(DPA)_3_] (in MeOH).

As mentioned above, the dipicolinic acid anion is a well‐established antenna for various luminescent lanthanoids.^[^ ^16^ ^]^ Beneficially, the high energy absorption band centered around 271 nm (36,900 cm^−1^) avoids interference with the switching wavelengths of most photoswitches. We chose diazocine as photoswitch to combine with this favorable antenna ligand.^[^ ^17^ ^]^ These “inverted azobenzenes” (see Figure 1) have a thermodynamically more stable *cis* state and provide the necessary switching state‐dependent absorptivity in the UV. Their switching wavelengths are in the visible part of the spectrum, avoiding overlap with the absorption band of the dipicolinic acid antenna. For example, the absorption maxima of the switching n─π* transition of the unfunctionalized diazocine are at 401 nm (*cis* state) and 483 nm (*trans* state), respectively, so that indirect sensitization via dipicolinate excitation should not affect the photoswitch (see Figure 3).

To estimate the extent of photomodulation realizable with the competitive absorption strategy in samples combining dipicolinate as antenna and diazocine as photoswitch, we determined the extinction coefficients of Na_3_[Eu(DPA)_3_] as well as diazocine at both photostationary states (PSSs) (see Supporting Information for details). From these we deduced a small but significant effect of the state of the photoswitch on the luminescence intensities of Na_3_[Ln(DPA)_3_]‐complexes, based on the competitive absorption mechanism (Table 1). These effects should be the minimum effect observable with any lanthanoid at a given relative concentration of diazocine. For our initiating studies we chose Tb^III^, Eu^III^, and Yb^III^, the three most emissive luminescent lanthanoids in the VIS and NIR, respectively.

**Table 1:** Estimated effect of luminescence intensity realizable via the competitive absorption mechanism in samples containing Na_3_[Ln(DPA)_3_]‐complexes and varying equivalents of diazocine.

Equivalents of diazocine	1	3	5
Estimated effect (%)	15	30	35

**Figure 4:**
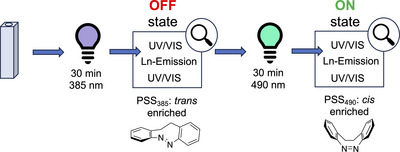
Procedure for room temperature irradiation experiments performed in this study.

From a practical perspective, a particular strength of the competitive absorption strategy is its independence of the spatial arrangement of the photophysically active components in the sample or material, i.e., no covalent connection between the lanthanoid complex and the photoswitch is required. To showcase the simplicity of this approach, we chose to demonstrate the principle with the simplest conceivable system based on DPA^2‐^ antennas and diazocine photoswitches: in situ prepared samples of Na_3_[Ln(DPA)_3_] and unfunctionalized diazocine (see Figure 1). In our photophysical experiments, all samples were initially irradiated with light of *λ* = 385 nm to reach the *trans*‐enriched PSS_385_ before steady‐state emission spectra and luminescence decays with indirect sensitization via the DPA^2−^ antenna ligand (*λ*_ex_ = 271 nm) were recorded. After exposure to light of *λ* = 490 nm, the respective experiments were performed analogously for the *cis*‐enriched PSS_490_. After each irradiation step and after each set of photoluminescence experiments, absorption spectra were recorded to monitor the state of the photoswitch. (Figure 4).

### 2.2 Photoswitching of Tb^III^ and Eu^III^ Luminescence

**Figure 5:**
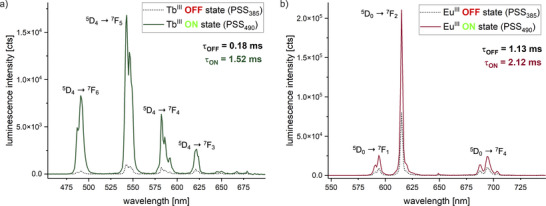
Emission spectra of samples containing Na_3_[Ln(DPA)_3_] (Ln^III^ = Tb^III^ (a) or Eu^III^ (b)) and five (Tb^III^) or three (Eu^III^) equivalents of diazocine at the OFF state (PSS_385_) and ON state (PSS_490_) (*c*(diazocine) = 2.5 or 1.5 mM, *c*(Ln^III^) = 0.5 mM, *c*(Na_2_DPA) = 1.5 mM, in MeOH, *λ*_ex_ = 271 nm).

We initiated our study by performing experiments on samples containing Tb^III^ or Eu^III^ and varying equivalents of diazocine. In line with the anticipated competitive absorption effect, we found that luminescence intensities were significantly lower at PSS_385_, where the photoswitch is more UV absorbent (see Figure 5 and Table 2). Hence, we will refer to the PSS reached after irradiation with *λ* = 385 nm as “OFF‐state” and to the PSS reached after irradiation with *λ* = 490 nm as “ON‐state”. Also, in line with expectations, we found that the observable effect on luminescence intensities was more pronounced for samples containing more diazocine.

**Table 2:** Absolute and relative differences of integrated luminescence intensities, observable monoexponential luminescence lifetimes (Tb^III^: *λ*_em_ = 543 nm, Eu^III^: *λ*_em_ = 615 nm) *τ*_OFF_ and *τ*_ON_ at the respective PSSs and change of luminescence lifetime Δ*τ* for in situ prepared samples containing LnCl_3_·6 H_2_O, Na_2_DPA and varying equivalents of diazocine upon indirect sensitization (*λ*_ex_ = 271 nm) at room temperature. *c*(Ln^III^) = 0.5 mM in MeOH. The OFF‐state refers to the system obtained after irradiation at 385 nm, the ON‐state refers to the system obtained after irradiation at 490 nm.

	Tb^III^, RT, *λ*_ex_ = 271 nm					Eu^III^, RT, *λ*_ex_ = 271 nm		
Equivalents of diazocine	0.25	1	3	5	0^[^ ^a^ ^]^	1	3	0^[^ ^a^ ^]^
Difference of integrated intensities **Δ*I*** [cts × λ]	1.51 × 10^6^	9.34 × 10^5^	2.60 × 10^5^	2.43 × 10^5^	—	1.45 × 10^6^	6.35 × 10^5^	—
**Relative difference** $\frac{\Delta I\cdot 100}{I_{\mathrm{ON}}}$ **(%)**	**49**	**80**	**87**	**93**	**—**	**53**	**61**	**—**
*τ*_OFF_ (ms)	1.35	0.56	0.26	0.18	1.7	1.71	1.13	2.28
*τ*_ON_ (ms)	2.02	1.26	1.22	1.52		2.22	2.12	
Δ*τ* (ms)	0.67	0.70	0.96	1.34	—	0.51	0.99	—
**Δ*τ* (%)**	**49**	**56**	**79**	**88**	**—**	**23**	**47**	**—**

^[a]^ pure Na_3_[Ln(DPA)_3_]

Effects were found to be more pronounced for samples containing Tb^III^ than for samples containing Eu^III^. In all samples containing these lanthanoids, the observed changes in luminescence intensity upon switching surpassed the effect due to pure competitive absorption as initially derived from extinction coefficients at 271 nm (see Table 1). The most pronounced effect of switching in our study was observed for the sample containing Tb^III^ and five equivalents of diazocine, where the experimentally determined switching effect of 93% is more than the 2.5‐fold of the effect expected to be due to competitive absorption at the excitation wavelength. This deviation is even higher for the sample containing Tb^III^ and one equivalent of diazocine (80% vs. 15%). Apart from the effect on photoluminescence intensities, we also observed a reversible shortening of luminescence lifetimes in the OFF states of our samples. These findings already gave first experimental evidence of additional effects contributing to the observed photomodulation of lanthanoid luminescence.

### 2.3 Additional effects

**Figure 6:**
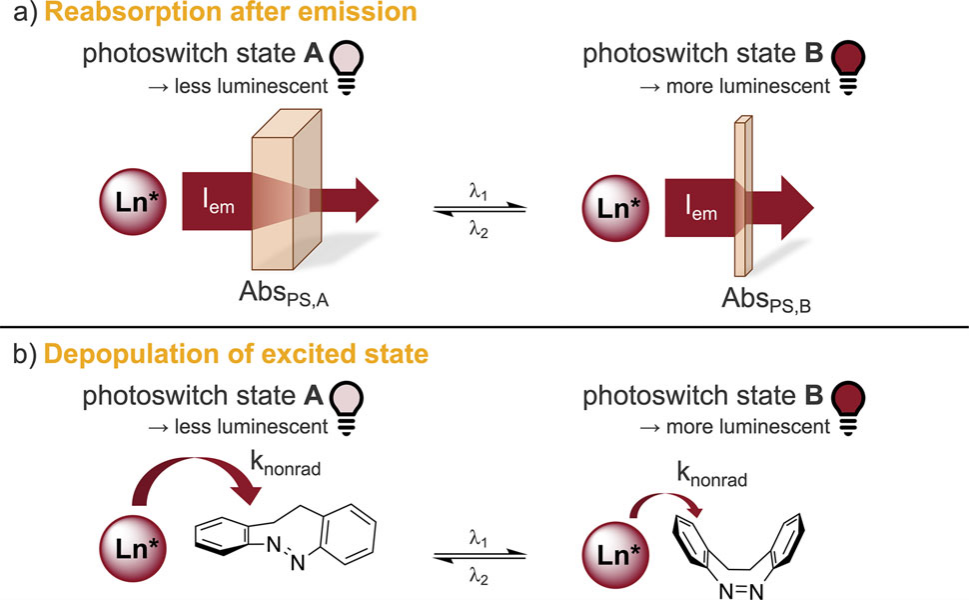
Further potential mechanisms for luminescence photoswitching. Luminescence photomodulation via a) reabsorption after emission and b) depopulation of excited state of the lanthanoid. Abs_PS,A_ and Abs_PS,B_ refer to the switching state absorptivity of the photoswitch. See main text for details.

The competitive absorption strategy targets the very first step of indirect sensitization via an antenna chromophore. However, also subsequent steps allow for a dynamic manipulation of the overall process (Figure 6).

Conceptually very similar to the competitive absorption is the “reabsorption after emission” mechanism (Figure 6a). A prerequisite for this process is a spectral overlap between the emission spectrum of the lanthanoid and the absorption spectrum of the photoswitch. If the spectral overlap changes upon switching of the photoswitch, this also results in an overall change of detectable lanthanoid luminescence since a varying amount of the photons emitted by the lanthanoid luminophore will be reabsorbed by the photoswitch. Comparison of the absorption spectra of diazocine in both states (see Figure 3) with the emission spectra of Tb^III^ and Eu^III^ (see Figure 5) reveals that such a process should be contributing at least in the case of Tb^III^.

A third potential contribution has a fundamentally different mechanism. This is the “depopulation of the excited state” (see Figure 6b) of the lanthanoid via nonradiative energy transfer onto a close‐range interaction partner. Such quenching processes are well studied for lanthanoid luminophores, and, for example, the reduction of energy transfer to O─H and C─H oscillators has been found to be a powerful tool to boost lanthanoid luminescence.^[^ ^18^ ^]^ If this process becomes photocontrollable, it can become another mechanism contributing to an overall photomodulation of lanthanoid luminescence. From the three mechanisms discussed herein, only the depopulation of the excited state can result in a photomodulation of the luminescence lifetimes, so from the data obtained at the beginning of our study, it was already very likely that such a depopulation of the excited state contributes to the overall observed effect.

**Figure 7:**
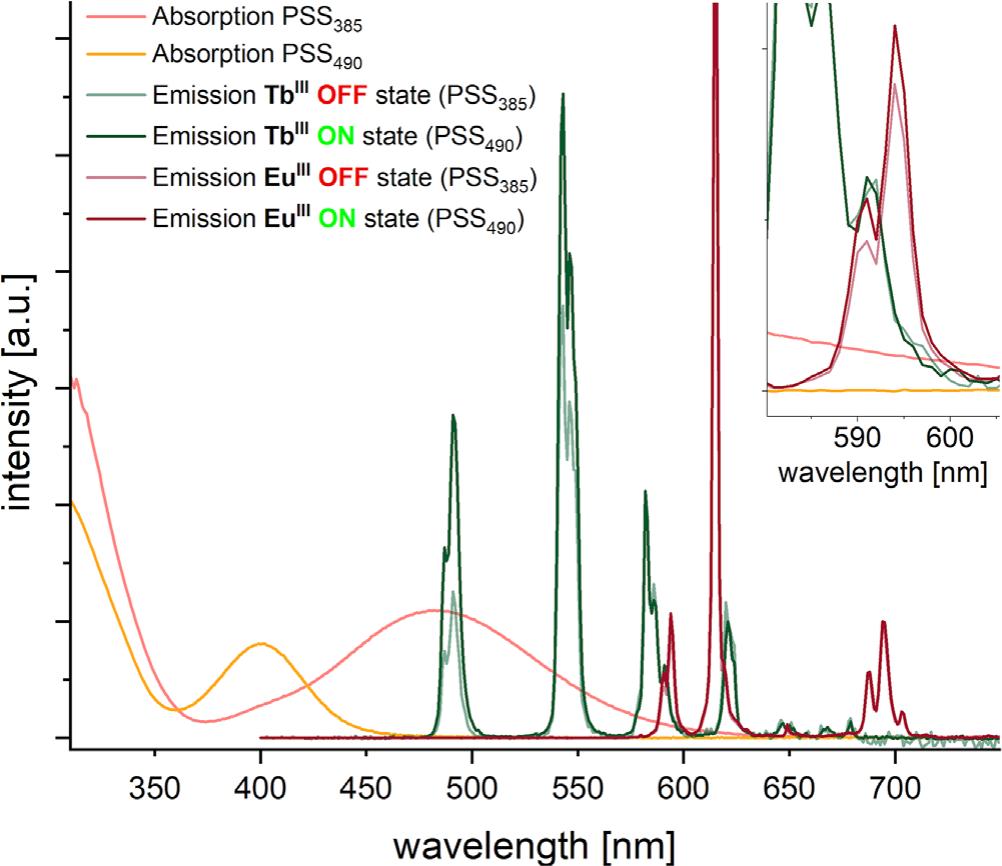
Absorption spectra of the sample containing Tb^III^ and three equivalents of diazocine and emission spectra of this sample and the analogous sample with Eu^III^, each at the ON‐state (PSS_385_) and OFF‐state (PSS_490_). Emission spectra of Tb^III^ were normalized for luminescence intensity at 621 nm, emission spectra of Eu^III^ were normalized for luminescence intensity at 694 nm. (*c*(diazocine) = 1.5 mM, *c*(Ln^III^) = 0.5 mM, *c*(Na_2_DPA) = 1.5 mM, in MeOH, *λ*_ex_ = 271 nm).

To access a potential contribution from a reabsorption after emission, we re‐examined the emission spectra obtained from our experiments with Tb^III^ and Eu^III^. For both lanthanoids, the shape and fine structure of the individual bands in the emission spectra was found to be invariant to the presence or switching state of the photoswitch.^[^ ^19^ ^]^ However, for samples with Tb^III^ it was observed that the intensity of higher energy transitions was affected more by the switching state of the photoswitch than the intensity of lower energy transitions. This becomes most evident in a plot of the emission spectra at both states, which have been normalized to the intensity of the respective lowest energy transition. As it can be seen in the respective plot for Tb^III^ in Figure 7, the emission intensities at 490 and 543 nm (^5^D_4_ ➝ ^7^F_6_ and ^5^D_4_ ➝ ^7^F_5_) were reduced more strongly upon photoswitching than the emission at 621 nm (^5^D_4_ ➝ ^7^F_3_), to which the spectra of both states were normalized. This correlates with the spectral overlap of the Tb^III^ emission with the absorption spectrum of the photoswitch at PSS_490_ and is evidence of the reabsorption by the photoswitch. Effectively, this relates to a red shift of Tb^III^ emission upon photoswitching, which is an uncommon observance in lanthanoid luminescence. Upon closer inspection, a similar change of relative intensities of transitions could be observed in the emission spectra of Eu^III^ samples. However, in line with the much smaller spectral overlap it was far less pronounced in this case (see insert in Figure 7).

Hence, considering the observable photomodulation of the luminescence lifetimes and the change of the relative intensities in the emission spectra, at least for the samples containing Tb^III^, all three mechanisms, competitive absorption, depopulation of the excited state, and reabsorption after emission, appear to be operational in the system under study.

### 2.4 Bypassing Competitive Absorption

We subsequently became interested in experiments which would allow us to access and potentially quantify individual contributions to the overall effect of photoswitching of lanthanoid luminescence. Absorption spectra recorded at both PSSs of the photoswitch revealed an isobestic point of the diazocene at *λ* = 361 nm (see Figure 3). Upon irradiation with light of this wavelength, the number of photons absorbed by the photoswitch will be independent of the switching state and it is possible to bypass the competitive absorption contribution to the overall effect. Initial experiments showed that for samples containing pure Na_3_[Tb(DPA)_3_] also after excitation with *λ*_ex_ = 361 nm (instead of *λ*_ex_ = 271 nm, which is the absorption maximum of the antenna, see Figure 3) the emission of Tb^III^ could be detected. Light of 361 nm is close in energy to the ^7^F_6_ ➝ ^5^D_3_ transition of Tb^III^, which is known to be fairly accessible to direct excitation.^[^ ^20^ ^]^ Excitation scans performed on samples of Na_3_[Tb(DPA)_3_] with or without the photoswitch at any PSS (see Supporting Information) revealed that under excitation at *λ*_ex_ = 361 nm for these samples a direct excitation can be expected to be somewhat contributing, however the main excitation pathway still appears to be via an organic antenna. We then repeated our previous experiments but changed the excitation wavelength from 271 to 361 nm).

For all samples studied, upon excitation at 361 nm, the observable effect of photoswitching upon the luminescence intensity dropped compared to the previous experiments (see Table 3). This is in line with expectations, since under these conditions the competitive absorption cannot contribute to the overall effect. Change of the excitation wavelength had a particularly pronounced effect for samples containing less than three equivalents of photoswitch. A plot of switching efficiency versus c(diazocine) (see Supporting Information) highlights a saturation effect for both excitation wavelengths at high concentrations of diazocine; for the sample with five equivalents of diazocine, the switching effect was almost the same for both excitation wavelengths. Since both competitive absorption and reabsorption after emission should be equally linearly dependent on the photoswitch concentration, this points to the depopulation of the excited state becoming particularly relevant for the overall switching effect at high concentrations of photoswitch.

**Table 3:** Relative difference of integrated luminescence intensities upon photoswitching, for in situ prepared samples containing TbCl_3_·6H_2_O, Na_2_DPA and varying equivalents of diazocine upon sensitization at *λ*_ex_ = 271 nm or sensitization at *λ*_ex_ = 361 nm, both at room temperature. *c*(Tb^III^) = 0.5 mM in MeOH. Relative differences of intensity were calculated analogously to Table 2.

Equivalents of diazocine	0.25	1	3	5
Relative difference $\frac{\Delta I\;\times \;100}{I_{\mathrm{ON}}}$ for ***λ*_ex_ = 271 nm** (%)	49	80	87	93
Relative difference $\frac{\Delta I\;\times \;100}{I_{\mathrm{ON}}}$ for ***λ*_ex_ = 361 nm** (%)	30	56	81	92
Δ (%)	19	24	6	1

### 2.5 Luminescence Lifetimes and Nonradiative Energy Transfer

**Figure 8:**
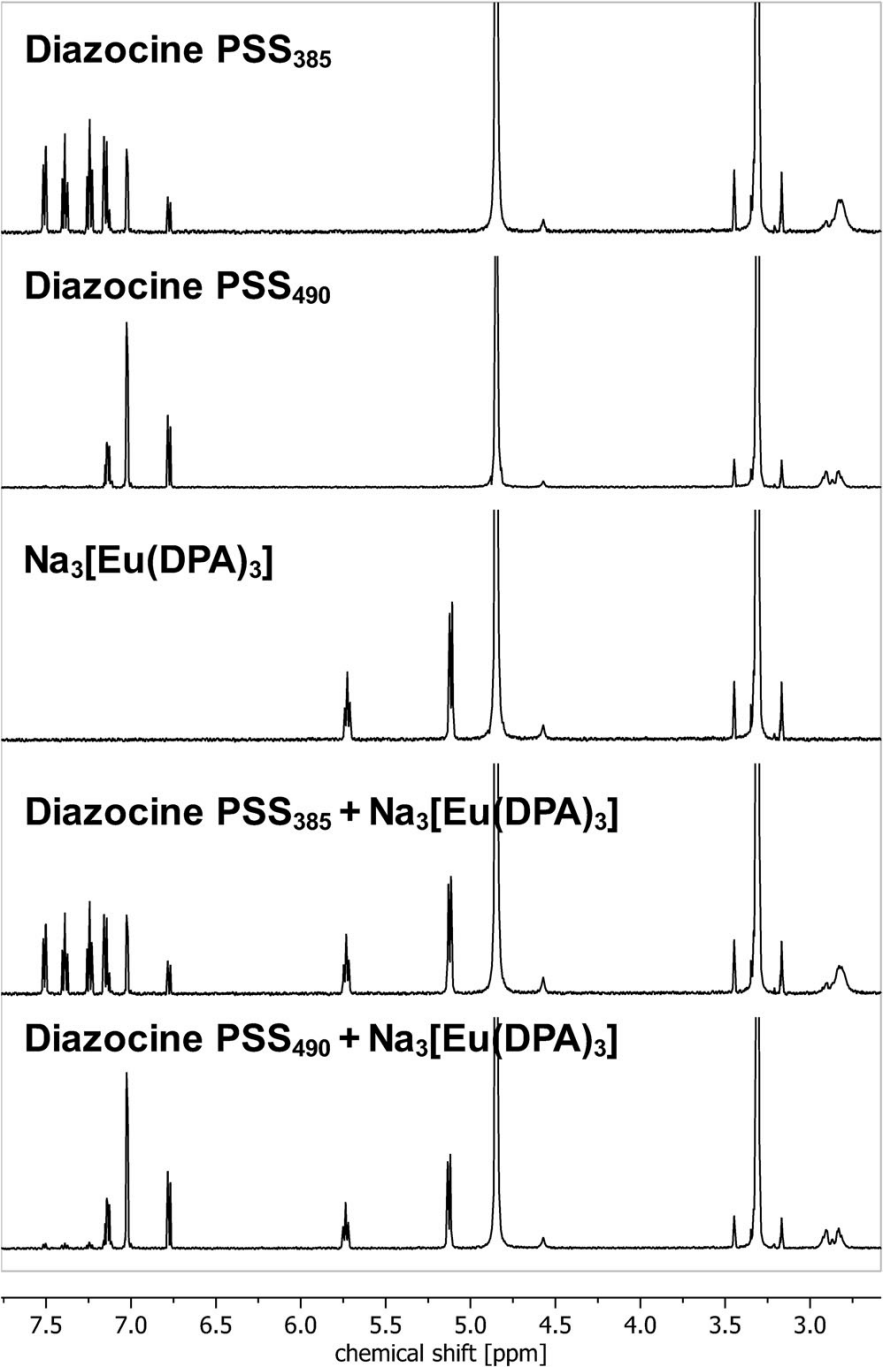
^1^H NMR spectra (500 MHz, CD_3_OD) of (from top to bottom): diazocine at PSS_385_, diazocine at PSS_490_, Na_3_[Eu(DPA)_3_], equimolar mixture of diazocine and Na_3_[Eu(DPA)_3_] at PSS_385_, equimolar mixture of diazocine and Na_3_[Eu(DPA)_3_] at PSS_490_.

For all samples containing Tb^III^ or Eu^III^, not only a change of the luminescence intensity was observed upon switching of the photoswitch, but also the observable luminescence lifetimes *τ*_obs_ were drastically affected. For the sample containing Tb^III^ and five equivalents of diazocine, the biggest effect on *τ*_obs_ was detected, with a total shortening of the luminescence lifetime of about 1.34 ms (*τ*_ON_ = 1.52 ms vs. *τ*_OFF_ = 0.18 ms, 88%; see Table 2). Of all three mechanisms discussed herein (see Figures 2 and 6), only a direct depopulation of the excited state via a nonradiative energy transfer can explain such a modification of *τ*_obs_. Nonradiative energy transfer processes are typically most efficient if the distance between donor and acceptor is small; for example, the efficiency of Förster‐type energy transfer processes^[^ ^21^ ^]^ is inversely dependent on the sixth power of the distance. Such a process would hence be expected to be most efficient with the photoswitch being in the first coordination sphere around the lanthanoid. Ligand field effects are very small for lanthanoid coordination compounds and typically only lead to subtle changes in their emission spectra. However, in the case of the highly symmetric complexes Na_3_[Ln(DPA)_3_], any close‐range interaction of the lanthanoid with alternative or additional ligands would likely be observable in the fine structure of the emission spectra. During our experiments, we found that the emission bands of our samples were indifferent to the presence of the photoswitch as well as its switching state. This strongly points to the first coordination sphere around the lanthanoid not being affected by the manipulation of the photoswitch and the photoswitch not being directly coordinated to the lanthanoid. We got complementary evidence for this conclusion from ^1^H NMR experiments in which spectra of samples containing the photoswitch with or without Na_3_[Eu(DPA)_3_] were recorded at PSS_385_ and PSS_490_, respectively (Figure 8).

Similarly to the effect of Eu^III^‐based paramagnetic shift reagents,^[^ ^22^ ^]^ a close‐range interaction of the diazocine and the Eu^III^‐ion would result in a change of the shifts of the signals corresponding to the protons of the diazocine. But in our case, shifts of both isomers of the photoswitch were found to be invariant to the presence of the paramagnetic lanthanoid complex. Concurrently, the signals of the DPA‐ligand are not affected by the presence of the photoswitch at either PSS.

These findings point to a scenario in which the species allowing for a nonradiative energy transfer between lanthanoid and photoswitch is only formed transiently as an exciplex‐type species.^[^ ^23^ ^]^ Indeed, if the resulting nonradiative energy transfer was efficient enough, the presence of exciplexes would not necessarily lead to an observable variation of the band shapes in the steady‐state emission spectra. Similarly, if these species were short‐lived enough or only present in the excited state, they would not be detectable via NMR. However, in solution‐state samples of the concentrations used herein, exciplex formation requires fast diffusion of components. To further study the nature of the nonradiative energy process, we next studied our samples in frozen solution (MeOH/EtOH 1:1) at 77 K. Under these conditions, any processes requiring diffusion should be strongly suppressed, and if the photomodulation of the luminescence lifetime relied on an exciplex‐based mechanism, it should no longer be observable. To ensure comparability to the studies performed at room temperature, the samples were brought to the two PSSs by irradiation in standard cuvettes at room temperature. Aliquots of the samples were then mixed with EtOH (v%/v% = 50/50), transferred into a quartz EPR tube, and then frozen in liquid nitrogen. As expected, in these experiments for Eu^III^ as well as Tb^III^, luminescence lifetimes *τ*_obs_ at 77 K were found to be independent of the state of the photoswitch, within error independent of the equivalents of diazocine and identical to *τ*_obs_ of samples containing no diazocine (see Table 4). This correlated with a significant drop of the observed switching effect upon the luminescence intensity (for example, from 53% to 5% for the sample containing Eu^III^ and one equivalent of diazocine and from 80% to 24% for the analogous sample with Tb^III^). At 77 K the major pathway leading to the reduction of luminescence intensity at the OFF state (PSS_385_) is suppressed.

**Table 4:** Relative difference of integrated luminescence intensities upon photoswitching, observable monoexponential luminescence lifetimes (Tb^III^: *λ*_em_ = 543 nm, Eu^III^: *λ*_em_ = 615 nm) *τ*_OFF_ and *τ*_ON_ at the respective PSSs and change of luminescence lifetime Δ*τ* for in situ prepared samples containing LnCl_3 _· 6H_2_O, Na_2_DPA and varying equivalents of diazocine upon indirect sensitization (*λ*_ex_ = 271 nm) at 77 K. *c*(Ln^III^) = 0.5 mM in MeOH. Relative differences of Intensity were calculated analogously to Table 2.

	Tb^III^, 77 K, *λ*_ex_ = 271 nm					Eu^III^, 77 K, *λ*_ex_ = 271 nm		
Equivalents of diazocine	0.25	1	3	5	0^[^ ^a^ ^]^	1	3	0^[^ ^a^ ^]^
**Relative difference** $\frac{\Delta I\;\times \;100}{I_{\mathrm{ON}}}$ **(%)**	**2**	**24**	**64**	**64**	**—**	**5**	**34**	**—**
*τ*_OFF_ (ms)	1.83	1.83	1.76	1.68	1.85	2.25	2.24	2.26
*τ*_ON_ (ms)	1.83	1.83	1.78	1.71		2.25	2.25	

^[a]^ pure Na_3_[Ln(DPA)_3_]

**Figure 9:**
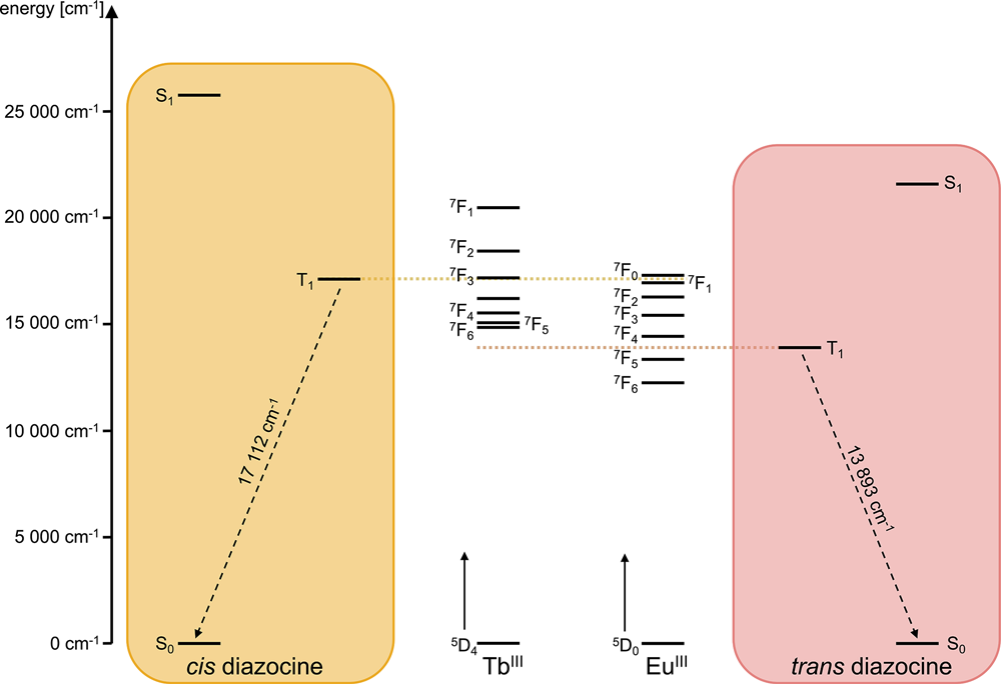
Calculated S_0_, S_1_, and T_1_ states of diazocine in the *cis* (left) and *trans* state (right) together with electronic energy levels of Tb^III^ and Eu^III^ as calculated by Carnall et al.^[^ ^24^ ^]^

While at room temperature the switching effect was more pronounced for the Tb^III^‐containing sample with five equivalents of diazocine than for the Tb^III^‐containing sample with only three equivalents of diazocine (93% vs. 87%, see Table 2), at 77 K an effect of only 64% was found for both samples. This reveals diffusion to be the limiting step under these conditions, pointing towards a relatively long‐lived accepting state being involved in the energy transfer onto the photoswitch. Searching for a suitable accepting state, we performed TD DFT calculations (wB97X‐D4/def2‐SVP with a conductor‐like polarizable continuum model for methanol) on both isomeric forms of the diazocine. This revealed a significant drop of the lowest triplet state of the diazocine upon switching (see Figure 9).

In the case of Tb^III^ this results in the energy gap between T_1_ and S_0_ of the photoswitch being smaller than the smallest energy gap of the lanthanoid (^5^D_4_ → ^7^F_6_) which can be expected to greatly facilitate the energy transfer. For Eu^III^, according to our calculations, the triplet of the *cis* state of diazocine is almost resonant with the weak ^5^D_0_ → ^7^F_0_‐transition which could not be observed in the samples studied herein. Upon switching, however, the energetic distance between T_1_ and S_0_ becomes smaller than the energy of the ^5^D_0_ → ^7^F_2_‐transition dominating the spectra. This makes the switching‐state dependent T_1_ of the diazocine a plausible candidate as accepting state in a nonradiative deactivation of Tb^III^ and Eu^III^.

We also repeated steady state emission experiments with excitation at 361 nm at 77 K. Under these conditions the competitive absorption at the excitation wavelength of the antenna is bypassed and quenching via diffusion dependent nonradiative energy transfer is suppressed. However, reabsorption of lanthanoid luminescence by the photoswitch could still occur. Considerable baseline correction before the integration of the resulting spectra was necessary and limits the interpretability of the data, yet a further reduction of the observed switching effect could be observed. For neither of the samples an effect surpassing 10% was detected under these conditions. Simple reabsorption after lanthanoid emission is hence the least important contribution to the overall switching effect upon lanthanoid luminescence in these systems. Nonradiative energy transfer, probably after exciplex formation, is much more relevant. Since this process is distance‐dependent this points to the importance of the control of spatial arrangement of photoactive components in the design of more elaborate, covalently connected systems for the photoswitching of lanthanoid luminescence.

### 2.6 Photoswitching Yb^III^ luminescence

**Figure 10:**
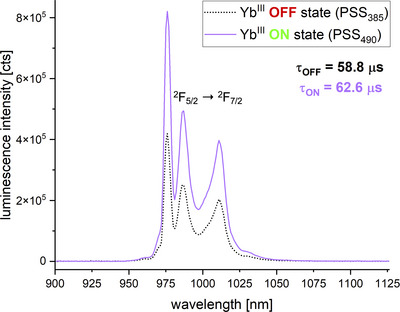
Emission spectrum of a sample containing Na_3_[Yb(DPA)_3_] and one equivalent of diazocine at the OFF state (PSS_385_) and ON state (PSS_490_) (*c*(diazocine) = 0.5 mM, *c*(Yb^III^) = 0.5 mM, *c*(Na_2_DPA) = 1.5 mM, in MeOH, at 77K, *λ*_ex_ = 271 nm).

After studying the most luminescent lanthanoids emitting in the visible part of the spectrum, we expanded our study to Yb^III^ which is considered the most robust NIR‐emitter of the trivalent lanthanoids (see Figure 10 and Table 5). Since Yb^III^ does not possess any electronic transitions in the visible part of the spectrum (the only excited state having an energy of approximately 10,250 cm^−1^),^[^ ^18^ ^]^ neither reabsorption by the photoswitch nor nonradiative energy transfer from the lanthanoid's excited state can contribute to a switching effect, and competitive absorption in the UV was anticipated to be the main origin of luminescence switching. 

**Table 5:** Percentual reduction of integrated luminescence intensity upon photoswitching, observable monoexponential luminescence lifetimes (*λ*_em_ = 974 nm) *τ*_OFF_ and *τ*_ON_ at the respective PSSs and change of luminescence lifetime Δ*τ* for in situ prepared samples containing YbCl_3_·6H_2_O, Na_2_DPA and varying equivalents of diazocine upon indirect sensitization (*λ*_ex_ = 271 nm) at room temperature and at 77 K. *c*(Yb^III^) = 0.5 mM in MeOH. Relative differences of intensity were calculated analogously to Table 2.

Equivalents of diazocine	1		3		0^[^ ^a^ ^]^	
	RT	77 K	RT	77 K	RT	77 K
**Relative difference** $\frac{\Delta I\;\times \;100}{I_{\mathrm{ON}}}$ **(%)**	**44**	**49**	**16**	**8**	—	—
*τ*_OFF_ (µs)	6.6	58.5	—^[^ ^b^ ^]^	61.8	6.9	69.0
*τ*_ON_ (µs)	6.6	62.6	—^[^ ^b^ ^]^	61.2		

^[a]^ pure Na_3_[Yb(DPA)_3_].

^[b]^ no reliable values for luminescence lifetimes of the sample with three equivalents of diazocine could be obtained at room temperature.

As expected, the luminescence intensity of the samples was rather weak at room temperature, and for interpretation of the integrated luminescence intensity a significant error of about ±10% has to be considered. Within these margins, for the Yb^III^‐containing samples with one or three equivalents of diazocine, the observable effect of photoswitching was found to be invariant to the temperature/phase of the sample. Equally, the observable luminescence lifetime *τ*_obs_ of the sample with three equivalents of diazocine was found to be invariant to the state of the photoswitch and, within error, equivalent to the value found for the reference sample without photoswitch. Hence there is no evidence for a nonradiative deactivation of the excited state contributing to the overall photomodulation of Yb^III^. Unlike for the samples with Eu^III^ and Tb^III^, for Yb^III^ the samples containing more equivalents of diazocine were found to exhibit a smaller effect of photoswitching. For the sample with one equivalent of photoswitch, an effect of approximately 44% was observed, while for the sample with three equivalents, only a value of about 16% was determined at room temperature. Due to the smaller energy gap between excited and ground states, NIR‐emitting lanthanoids are much more prone to nonradiative deactivation via multiphonon quenching than lanthanoids emitting in the VIS.^[^ ^18^ ^]^ At the same time, the energy gap between the feeding triplet state of the antenna ligand and the excited state of the lanthanoid is particularly large in the case of Yb^III^. Potentially, at higher concentrations of diazocine, processes related to these circumstances become dominant and overrule the effects described above for Eu^III^ and Tb^III^.

## 3 Conclusion

As we were able to show, with the aid of diazocine, the luminescence of lanthanoid dipicolinates can efficiently be photomodulated. Effects of up to 93% reduction of luminescence intensity and 88% (1.34 ms) reduction of luminescence lifetime were observed for Tb^III^, significant effects were also observed for Eu^III^ and Yb^III^. Importantly, in this system, the photomodulation of lanthanoid luminescence does not require covalent connection of antenna ligand and photoswitch or direct coordination of the photoswitch. This provides great freedom for the design of more elaborate systems incorporating more complex functionalities, for example in the context of multiplexing. Studies with an alternate excitation wavelength and in frozen solution at 77 K provided first insights into the contributions of three different mechanisms to the overall effect, which are competitive absorption, depopulation of the excited state and reabsorption after emission. Relative contributions depend on the nature of the lanthanoid. Future experiments including more specialized techniques such as transient absorption will contribute to a more detailed understanding of the radiative and nonradiative energy transfer processes leading to the observed effect.

## 4 Experimental Section

### Synthesis and sample preparation

Dipicolinic acid (DPA), Sodium hydroxide (NaOH), Lanthanoid trichlorides (LnCl_3_· 6H_2_O), chemicals and solvents (HPLC grade) required for synthesis were purchased and used without further purification. Na_3_[Eu(DPA)_3_]^[^ ^25^ ^]^ was prepared according to a previously reported procedure and diazocine^[^ ^26^ ^]^ was prepared according to a modified previously reported procedure (see supporting information for details). Column chromatography was performed using silica gel 60 (Merck, 0.063‐0.200 mm). Analytical thin layer chromatography (TLC) was performed with silica gel 60 F_254_ plates (Merck, coated on aluminum sheets). All luminescence studies were performed in HPLC grade MeOH or 1:1 v%/v% mixtures with HPLC grade EtOH, all NMR studies were performed in CD_3_OD. Samples were prepared directly before use from stock solutions.

### Irradiation of samples

Samples were irradiated with 385 nm (bandwidth (FWHM) 10 nm, output power > 9.0 mW) or 490 nm (bandwidth (FWHM) 26 nm, output power > 2.3 mW) LEDs purchased from Thorlabs. A T‐Cube LED Driver (1200 mA Max Drive Current) was used. To control the spatial arrangement of sample and LED, a 3D printed cuvette holder and the LED were screwed upon an optical plate (see Supporting Information for a photograph of the setup). The complete setup was placed inside a home‐built cabinet during irradiation. For initial switching to the PSSs samples were irradiated for 30 min. For room temperature samples, after steady state emission experiments and prior to recording of luminescence decay profiles samples were irradiated for another 5 min.

### Photophysical measurements

Samples were irradiated and studied (for experiments at room temperature) in rectangular 1 cm pathlength cuvettes (quartz suprasil). Emission spectra were recorded on an Edinburgh Instrument FLS1000 spectrometer, equipped with a 450 W Xenon arc lamp. Emission was collected at a 90° angle. For VIS‐detection a red‐sensitive photomultiplier (PMT‐980) was used, for NIR‐detection a N_2_‐cooled NIR photomultiplier (PMT‐1400) was used. For recording of luminescence decay profiles, a µF2 pulsed 60 W Xenon microsecond flashlamp with a repetition rate of 10 Hz was used together with a multichannel scaling (MSC) module. For measurements at 77 K an EPR Dewar filled with liquid nitrogen was employed. Here, after irradiation, methanolic samples were combined with identical volumes of EtOH and transferred into quartz EPR tubes prior to freezing. Absorption spectra were recorded with the aid of a silicon photodiode mounted within the sample chamber. For measurements at room temperature prior and after each set of measurements absorption spectra were recorded to monitor the state of the photoswitch.

### NMR measurements

NMR spectra were recorded on a Bruker AV 500 Avance NEO spectrometer. Chemical shifts are given in ppm and relative to residual proton signals of the solvent. Samples were irradiated in the NMR tubes directly before the measurements.

### DFT calculations

Structural optimizations and TD DFT calculations (wB97X‐D4/def2‐SVP/CPCM(methanol)) were performed with ORCA Program Version 5.0.1.^[^ ^27^ ^]^ Structures were first optimized with values of the diazocine dihedral CNNC being fixed to values from crystallographic data (*cis*: 0.7°^[^ ^17^ ^]^ and *trans* 148.2°^[^ ^28^ ^]^). Constraints were removed in a second optimization step upon which the structures remained in their local minima before singlets and triplets were calculated with a subsequent TD DFT calculation.

## Supporting Information

Synthetic details, information of the determination of extinction coefficients, plot of observed saturation effect of switching efficiency, photograph of irradiation setup (PDF).

## Conflicts of Interest

The authors declare no conflicts of interest.

## Supporting information



Supporting Information

## Data Availability

The data that support the findings of this study are available from the corresponding author upon reasonable request.

## References

[1] a) G. C. Thaggard , J. Haimerl , K. C. Park , J. Lim , R. A. Fischer , B. K. P. Maldeni Kankanamalage , B. J. Yarbrough , G. R. Wilson , N. B. Shustova , J. Am. Chem. Soc. 2022, 144, 23249; 36512744 10.1021/jacs.2c09879

[2] a) M. Olesinska‐Monch , C. Deo , Chem. Commun. 2023, 59, 660; 10.1039/d2cc05870g 36622788

[3] a) J.‐C. G. Bünzli , Coord. Chem. Rev. 2015, 293–294, 19;

[4] a) T. Lövgren , H. Mikola , I. Hemmilä , K. Blomberg , K. Pettersson , Y. Y. Xu , Clin. Chem. 1992, 3z8, 2038; 1394988

[5] Y. Fréroux , L. Caussin , N. El Beyrouti , S. Rigaut , L. Norel , in Including Actinides Women's Contribution to f‐element Science, Part 1 (Eds: J.‐C. G. Bünzli , S. M. Kauzlarich ), 2024, pp. 35.

[6] a) T. Nakagawa , K. Atsumi , T. Nakashima , Y. Hasegawa , T. Kawai , Chem. Lett. 2007, 36, 372;

[7] K. Machitani , Y. Nakahara , K. Kimura , Bull. Chem. Soc. Jpn. 2009, 82, 472.

[8] H. J. Yu , H. Wang , F. F. Shen , F. Q. Li , Y. M. Zhang , X. Xu , Y. Liu , Small 2022, 18, 2201737. 10.1002/smll.202201737 35585680

[9] Y. Zhou , H.‐Y. Zhang , Z.‐Y. Zhang , Y. Liu , J. Am. Chem. Soc. 2017, 139, 7168. 28509539 10.1021/jacs.7b03153

[10] N. Wang , J. Wang , D. Zhao , S. K. Mellerup , T. Peng , H. Wang , S. Wang , Inorg. Chem. 2018, 57, 10040. 30070839 10.1021/acs.inorgchem.8b01209

[11] W. L. Zhou , X. Y. Dai , W. Lin , Y. Chen , Y. Liu , Chem. Sci. 2023, 14, 6457. 37325139 10.1039/d3sc01425h PMC10266474

[12] a) H. Al Sabea , L. Norel , O. Galangau , H. Hijazi , R. Metivier , T. Roisnel , O. Maury , C. Bucher , F. Riobe , S. Rigaut , J. Am. Chem. Soc. 2019, 141, 20026; 31820955 10.1021/jacs.9b11318

[13] J. Xie , T. Wu , X. Wang , C. Yu , W. Huang , D. Wu , Inorg. Chem. 2020, 59, 15460. 32990428 10.1021/acs.inorgchem.0c02488

[14] a) A. K. R. Junker , L. R. Hill , A. L. Thompson , S. Faulkner , T. J. Sorensen , Dalton Trans. 2018, 47, 4794; 29560975 10.1039/C7DT04788F

[15] E. B. Sveshnikova , P. A. Shakhverdov , T. A. Shakhverdov , V. E. Lanin , R. U. Safina , B. M. Bolotin , V. L. Ermolaev , Opt. Spectrosc. 2003, 95, 898.

[16] a) H. G. Brittain , Inorg. Chem. 2002, 17, 2762;

[17] R. Siewertsen , H. Neumann , B. Buchheim‐Stehn , R. Herges , C. Nather , F. Renth , F. Temps , J. Am. Chem. Soc. 2009, 131, 15594. 19827776 10.1021/ja906547d

[18] E. Kreidt , C. Kruck , M. Seitz , Handbook on the Physics and Chemistry of Rare Earths, Elsevier, North‐Holland, 2018.

[19] K. Binnemans , Coord. Chem. Rev. 2015, 295, 1.

[20] M. E. Thornton , J. Hemsworth , S. Hay , P. Parkinson , S. Faulkner , L. S. Natrajan , Front. Chem. 2023, 11, 1232690. 37583568 10.3389/fchem.2023.1232690 PMC10424921

[21] a) T. Förster , Ann. Phys. 1948, 437, 55;

[22] J. K. M. Sanders , D. H. Williams , Nature 1972, 240, 385.

[23] F. Kielar , C. P. Montgomery , E. J. New , D. Parker , R. A. Poole , S. L. Richardson , P. A. Stenson , Org. Biomol. Chem. 2007, 5, 2975. 17728864 10.1039/b709062e

[24] a) W. T. Carnall , P. R. Fields , K. Rajnak , J. Chem. Phys. 1968, 49, 4447;

[25] G. Kervern , A. D'Aleo , L. Toupet , O. Maury , L. Emsley , G. Pintacuda , Angew. Chem., Int. Ed. 2009, 48, 3082. 10.1002/anie.200805302 19301347

[26] a) W. Moormann , D. Langbehn , R. Herges , Synthesis 2017, 49, 3471; 10.3762/bjoc.15.68 PMC6444418 30992720

[27] F. Neese , Wiley Interdiscip. Rev.:Comput. Mol. Sci. 2022, 12, e1606.

[28] R. Krämer , N. Nöthling , C. W. Lehmann , F. Mohr , M. W. Tausch , ChemPhotoChem 2017, 2, 6.

