# Supplementary material for: Photoswitching Lanthanoid Luminescence With Diazocines
Source: Chemistry. 2025 Apr 21;31(32):e202500397. doi: 10.1002/chem.202500397 (PMC12144903; doi:10.1002/chem.202500397)
Supplement: Supplementary file 1 — Supporting Information [file CHEM-31-e202500397-s001.pdf]

# Photoswitching Lanthanoid Luminescence with Diazocines

Nicole Winterholler, Elisabeth Kreidt*

TU Dortmund University, Faculty of Chemistry and Chemical Biology, Inorganic Chemistry, Otto-Hahn-Straße 6, 44227 Dortmund, Germany

Email: elisabeth.kreidt@tu-dortmund.de

# Supporting Information

### Table of contents

**Synthesis of 11,12-Dihydrodibenzo[c,g][1,2]diazocine** 2

**Determination of extinction coefficients** 3

**Integrated luminescence intensities of samples containing $Tb^{III}$ and $Eu^{III}$ at room temperature** 5

**Excitation Scans of samples containing $Tb^{III}$ and $Eu^{III}$** 6

**Saturation effect of switching efficiency in dependence of the concentration of diazocine** 7

**Evolution of absorbance in the visible range during repetitive switching cycles** 8

**Irradiation setup** 9

**References** 10

# Synthesis of 11,12-Dihydrodibenzo[c,g][1,2]diazocine

The diazocine was prepared via a modified version of a procedure described by the group of Herges[1]:

1,2-Bis(2-nitrophenyl)ethane ($C_{14}H_{12}N_2O_4$, 1.00 g, 272.26 g mol$^{-1}$, 3.67 mmol, 1 eq.) and barium hydroxide ($Ba(OH)_2$, 3.48 g, 315 g mol$^{-1}$, 11.0 mmol, 3 eq.) were suspended in a mixture of 200 mL of ethanol and 100 mL of water. The stirred mixture was brought to reflux and after 45 minutes zinc powder (Zn, 7.45 g, 65.38 g mol$^{-1}$, 113.86 mmol, 31 eq.) was added. After additional 6 hours at reflux temperature the mixture was filtered of celite and the solvent was removed under reduced pressure. A mixture of 200 mL of ethanol and 100 mL of water was added to the solid and the resulting mixture was brought to reflux temperature. Again barium hydroxide ($Ba(OH)_2$, 3.48 g, 315 g mol$^{-1}$, 11.0 mmol, 3 eq.) and zinc powder (Zn, 3.84 g, 65.38 g mol$^{-1}$, 113.86 mmol, 16 eq.) were added to the stirred solution. After 5 hours at reflux temperature the mixture was stirred for two days at room temperature and then for 5 more hours at reflux temperature before the mixture was filtered over celite and evaporated to dryness. The resulting solid was taken up in dichloromethane and again filtered over celite before the solvent was removed under reduced pressure. After column chromatography ($SiO_2$, cyclohexane:EtOAc 9:1 → 4:1) the product 11,12-dihydrodibenzo[c,g][1,2]diazocine ($C_{14}H_{12}N_2$, 196 mg, 208.26 g mol$^{-1}$, 1.16 mmol, 32%) was isolated as yellow solid.

## Determination of extinction coefficients

Extinction coefficients of the photoswitch and $Na_3[Eu(DPA)_3]$ were determined in MeOH, using samples of different concentrations and quartz suprasil cuvettes of path length 1 cm, 2 mm and 1 mm, respectively. For the determination of extinction coefficients of diazocine at $PSS_{385}$ and $PSS_{490}$ samples were irradiated for 30 minutes in the same setup that was used for the luminescence studies. Details of individual measurements are summarised below. Extinction coefficients were extracted as slope of the plot $E_{271nm}$ vs c*d (Figure S1):

Diazocine: $\varepsilon(271\ \text{nm}, PSS_{385}) = 3.18 \cdot 10^2\ m^2/mol$

$\varepsilon(271\ \text{nm}, PSS_{490}) = 1.55 \cdot 10^2\ m^2/mol$

$Na_3[Eu(DPA)_3]$: $\varepsilon(271\ \text{nm}) = 7.3 \cdot 10^2\ m^2/mol$

**Table S1:** Data used for determination of extinction coefficients of diazocine.

| **Diazocine** | | | | | |
|---|---|---|---|---|---|
| Sample | 1 | 2 | 3 | 4 | 5 |
| Concentration c [$10^{-3}$ mol/L] | 0.5 | 0.5 | 1.5 | 0.5 | 0.4 |
| Path length d [mm] | 1 | 2 | 1 | 10 | 10 |
| c*d [$10^{-3}$ mol*$m^{-2}$] | 0.5 | 1 | 1.5 | 5 | 4 |
| $E_{271nm}$ at $PSS_{385}$ | 0.1628 | 0.3231 | 0.4929 | 1.592 | 1.291 |
| $E_{271nm}$ at $PSS_{490}$ | 0.0878 | 0.1627 | 0.2554 | 0.7827 | 0.6384 |

**Table S2:** Data used for determination of extinction coefficients of $Na_3[Eu(DPA)_3]$.

| **$Na_3[Eu(DPA)_3]$** | | | | | | |
|---|---|---|---|---|---|---|
| Sample | 1 | 2 | 3 | 4 | 5 | 6 |
| Concentration c [$10^{-3}$ mol/L] | 0.5 | 0.167 | 0.017 | 0.5 | 0.25 | 0.2 |
| Path length d [mm] | 1 | 10 | 10 | 2 | 1 | 10 |
| c*d [$10^{-3}$ mol*$m^{-2}$] | 0.5 | 1.67 | 0.167 | 1 | 0.25 | 2 |
| $E_{271nm}$ | 0.4086 | 1.264 | 0.1366 | 0.7862 | 0.2068 | 1.460 |

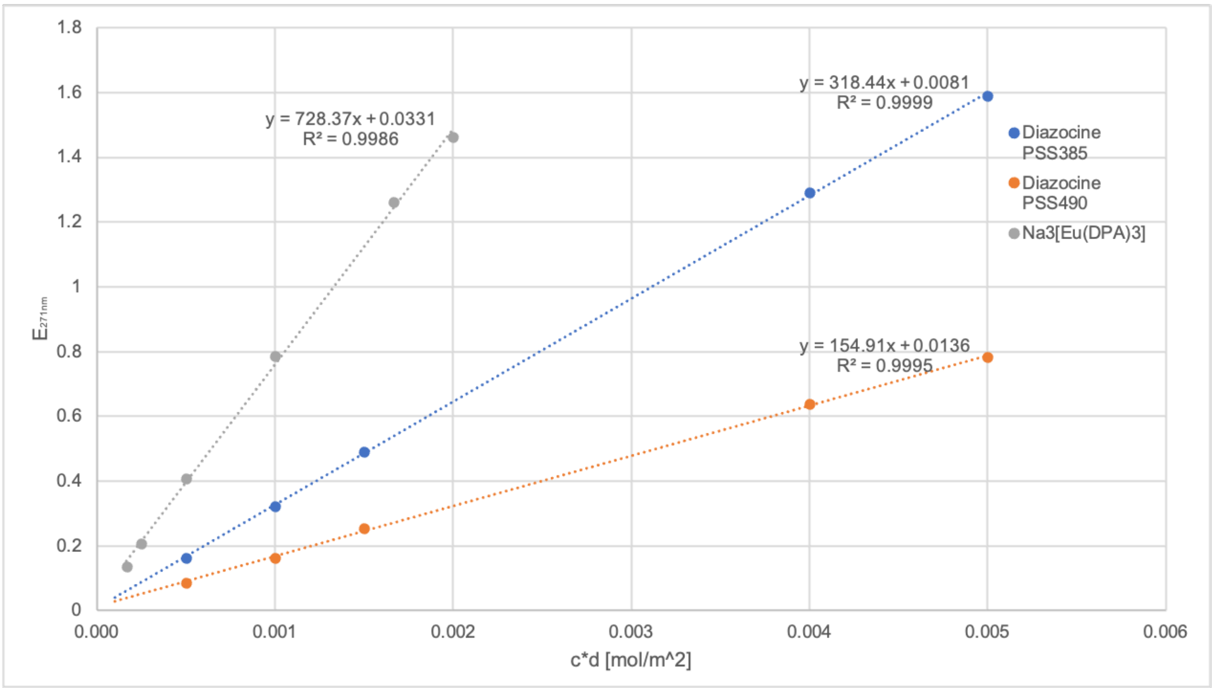


**Figure S1:** Plots for the determination of the extinction coefficients of diazocine and $Na_3[Eu(DPA)_3]$.

# Integrated luminescence intensities of samples containing $Tb^{III}$ and $Eu^{III}$ at room temperature

**Table S3.** Integrated luminescence intensities at both states for *in situ* prepared samples containing $LnCl_3 \cdot 6\ H_2O$, $Na_2DPA$ and varying equivalents of diazocine upon indirect sensitisation ($\lambda_{ex}$ = 271 nm) at room temperature. $c(Ln^{III})$ = 0.5 mM in MeOH.

| | **$Tb^{III}$**, RT, $\lambda_{ex}$ = 271 nm | | | | | **$Eu^{III}$**, RT, $\lambda_{ex}$ = 271 nm | | |
|---|---|---|---|---|---|---|---|---|
| Equivalents of diazocine | 0.25 | 1 | 3 | 5 | 0[a] | 1 | 3 | 0[a] |
| Integrated luminescence **$I_{OFF}$** intensity at **$PSS_{385}$**, [cts•λ] | $1.55 \cdot 10^6$ | $2.36 \cdot 10^5$ | $3.86 \cdot 10^4$ | $1.80 \cdot 10^4$ | $2.52 \cdot 10^6$ | $1.30 \cdot 10^6$ | $3.98 \cdot 10^5$ | $5.52 \cdot 10^6$ |
| Integrated luminescence **$I_{ON}$** intensity at **$PSS_{490}$** [cts•λ] | $3.06 \cdot 10^6$ | $1.17 \cdot 10^6$ | $2.99 \cdot 10^5$ | $2.61 \cdot 10^5$ | | $2.75 \cdot 10^6$ | $1.03 \cdot 10^6$ | |

[a] pure $Na_3[Ln(DPA)_3]$

# Excitation Scans of samples containing $Tb^{III}$ and $Eu^{III}$

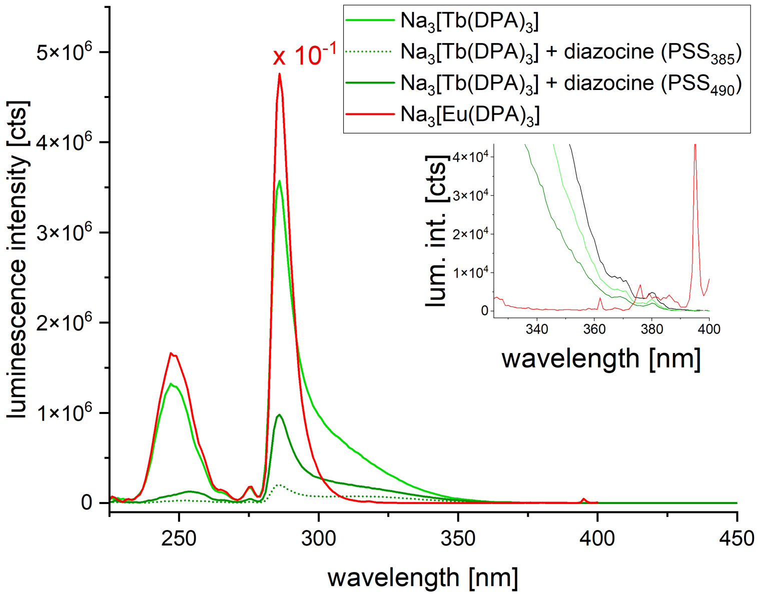


**Figure S2**: Excitation scans of $Na_3[Eu(DPA)_3]$, $Na_3[Tb(DPA)_3]$ or $Na_3[Tb(DPA)_3]$ with diazocince (0.5 mM) at either PSS in MeOH at room temperature ($c(Ln^{III})$ = 0.5 mM, $c(Na_2DPA)$ = 1.5 mM). For samples containing $Tb^{III}$ emission was monitored at 543 nm, for $Eu^{III}$ emission was recorded at 615 nm. The spectrum of $Na_3[Eu(DPA)_3]$ was multiplied with a factor of 0.1 to improve comparability of the qualitative features.

# Saturation effect of switching efficiency in dependence of the concentration of diazocine

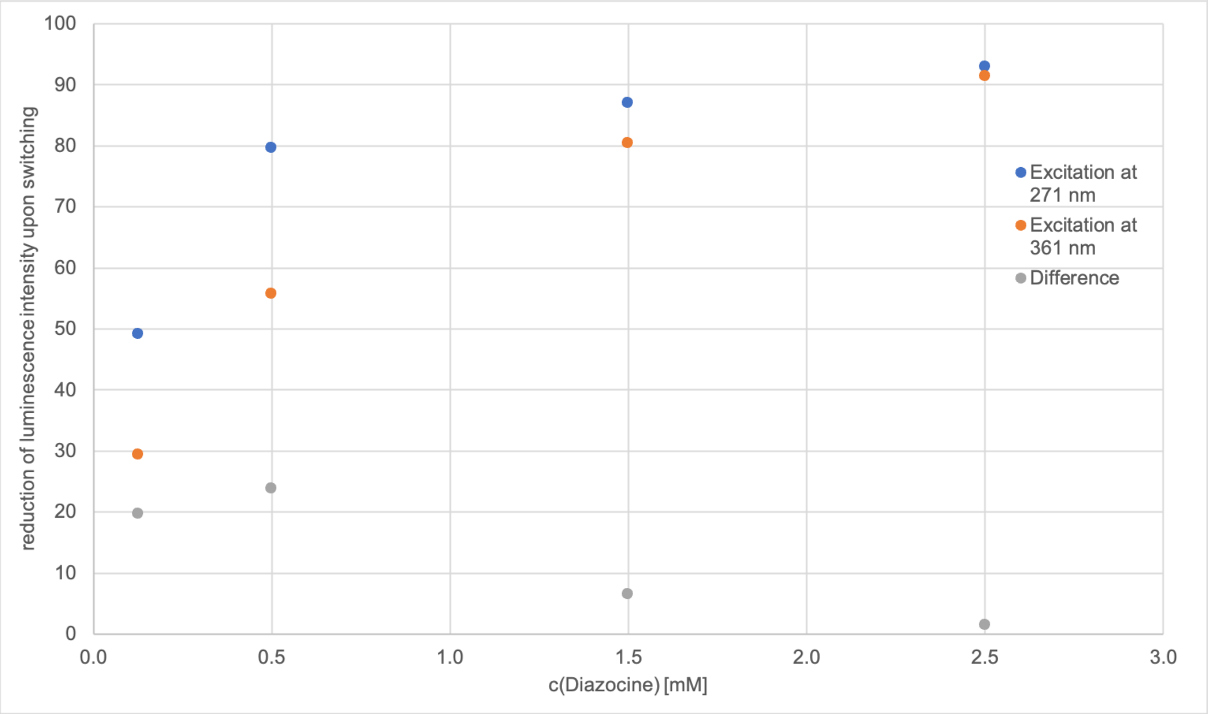


**Figure S3:** Reduction of luminescence intensity of $Tb^{III}$-containing samples with different concentrations of diazocine observed during luminescence experiments with excitation wavelengths $\lambda_{ex}$ = 271 nm or $\lambda_{ex}$ = 361 nm.

**Table S4.** Data used for figure S3.

| **Sample** | **1** | **2** | **3** | **4** |
|---|---|---|---|---|
| Concentration c [$10^{-3}$ mol/L] | 0.125 | 0.5 | 1.5 | 2.5 |
| Relative difference $\frac{\Delta I \cdot 100}{I_{ON}}$ for $\lambda_{ex}$ **= 271 nm** [%] | 49.3 | 79.8 | 87.1 | 93.1 |
| Relative difference $\frac{\Delta I \cdot 100}{I_{ON}}$ for $\lambda_{ex}$ **= 361 nm** [%] | 29.5 | 55.9 | 80.5 | 91.5 |
| Difference [%] | 19.8 | 23.9 | 6.6 | 1.6 |

# Evolution of absorbance in the visible range during repetitive switching cycles

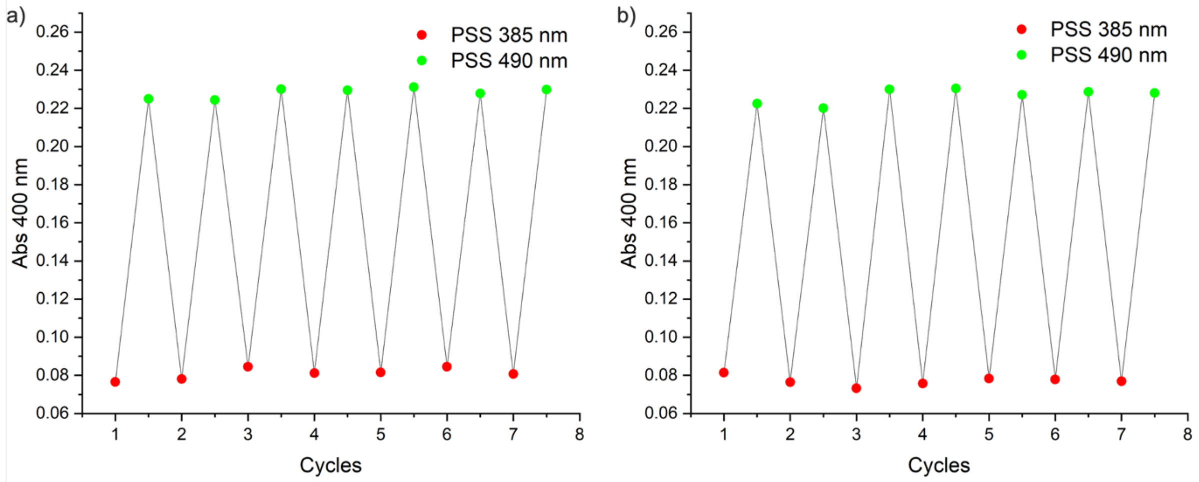


**Figure S4:** Evolution of the absorption at 400 nm during repetitive switching cycles for a sample of a) pure diazocine (0.5 mM) in MeOH or b) $Na_3[Tb(DPA)_3]$ ($c(Tb^{III})$ = 0.5 mM, $c(Na_2DPA)$ = 1.5 mM) and diazocine (0.5 mM) in MeOH. Irradiation time per cycle: 30 min $\lambda$ = 385 nm, 30 min $\lambda$ = 490 nm.

# Irradiation setup

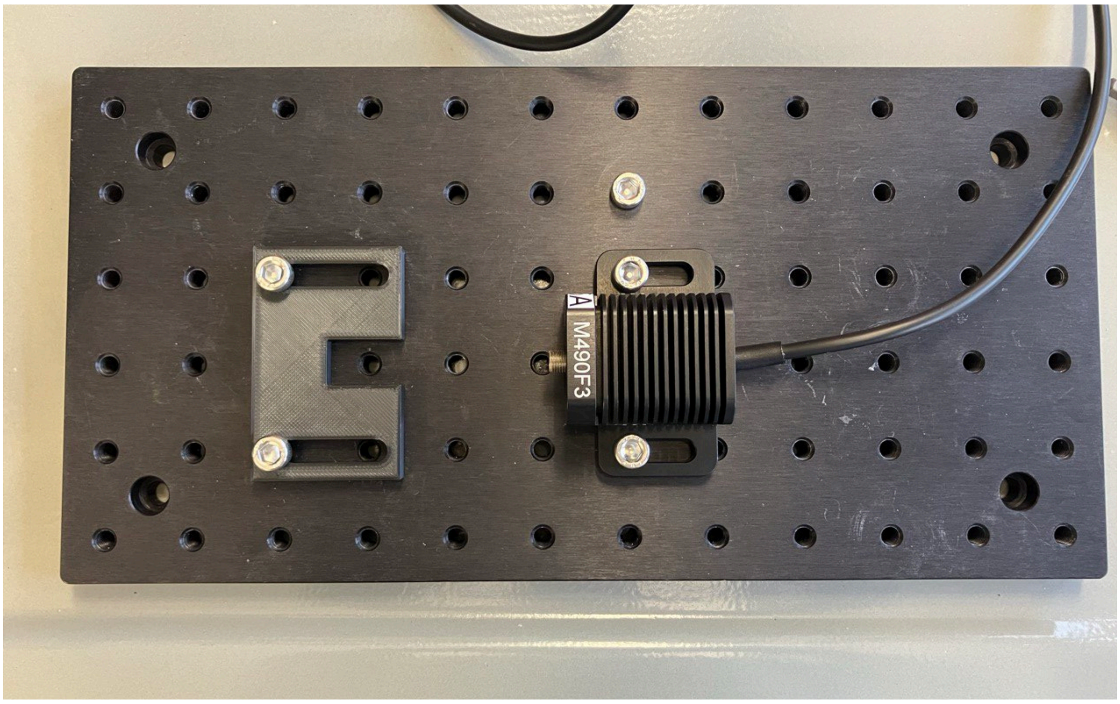


**Figure S5:** Photograph of the irradiation setup with LED screwed onto an optical plate. Cuvettes were placed inside the cavity of the 3D printed stencil; NMR tubes were placed in the hole in the cavity during irradiation.

# References

[1] a) W. Moormann, D. Langbehn, R. Herges, *Synthesis* **2017**, *49*, 3471-3475; b) J. Isokuortti, T. Griebenow, J. S. von Glasenapp, T. Raeker, M. A. Filatov, T. Laaksonen, R. Herges, N. A. Durandin, *Chem. Sci.* **2023**, *14*, 9161-9166.
